# Detection of two synchronous histologically different renal cell carcinoma subtypes in the same kidney: a case report and review of the literature

**DOI:** 10.1186/s13256-024-04527-x

**Published:** 2024-05-18

**Authors:** Mohamed Sakr, Merhan Badran, Sarah Ahmed Hassan, Mohamed Elsaqa, Mohamed Anwar Elwany, Nevine M. F. El Deeb, Mohamed Sharafeldeen

**Affiliations:** Faculty of Medicine, Alexandria University, Champollion Street, Alexandria, Egypt

**Keywords:** Clear cell renal cell carcinoma (ccRCC), Chromophobe renal cell carcinoma (ChRCC), Synchronous renal cell carcinoma, Different histology, Case report

## Abstract

**Introduction:**

Renal cell carcinoma (RCC) is the dominant primary renal malignant neoplasm, encompassing a significant portion of renal tumors. The presence of synchronous yet histologically distinct ipsilateral RCCs, however, is an exceptionally uncommon phenomenon that is rather under-described in the literature regarding etiology, diagnosis, management, and later outcomes during follow-up.

**Case presentation:**

We aim to present the 9th case of a combination chromophobe RCC (ChRCC) and clear cell RCC (ccRCC) in literature, according to our knowledge, for a 69-year-old North African, Caucasian female patient who, after complaining of loin pain and hematuria, was found to have two right renal masses with preoperative computed tomography (CT) and underwent right radical nephrectomy. Pathological examination later revealed the two renal masses to be of different histologic subtypes.

**Conclusion:**

The coexistence of dissimilar RCC subtypes can contribute to diverse prognostic implications. Further research should focus on enhancing the complex, yet highly crucial, preoperative detection and pathological examination to differentiate multiple renal lesions. Planning optimal operative techniques (radical or partial nephrectomy), selecting suitable adjuvant regimens, and reporting long-term follow-up outcomes of patients in whom synchronous yet different RCC subtypes were detected are of utmost importance.

## Introduction

The simultaneous coexistence of renal cell carcinoma (RCC) with other renal neoplasms is a rare occurrence. Literature has described many cases of benign tumors occurring synchronously with RCC in the same kidney. Even more uncommon is the presence of multiple different RCC subtypes as separate tumors or sometimes even within a single tumor [1–5]**.** This simultaneous occurrence of different RCC subtypes poses challenges in preoperative detection, operative techniques, postoperative follow-up, and adjuvant regimens. In this case report, we present a unique instance of synchronous clear cell renal cell carcinoma (ccRCC) and chromophobe renal cell carcinoma (ChRCC), contributing to the existing literature on this subject. We also review similar cases reported previously, discuss and compare methods for preoperative diagnosis of multifocal RCCs and their differentiation, explore operative approaches, and address challenges in the postoperative period and prognosis estimation.

Due to the rarity of this condition, there is a lack of literature reports detailing both its preoperative course, including detection and differentiation, and its postoperative course regarding adjuvant therapy and long-term follow-up. Therefore, we aim to present this case and review similar cases in the literature to underscore the urgent need for further research in this area.

## Case presentation

A 69-year-old North African Caucasian female patient presented to the Urology department at Alexandria Medical Center, Egypt, with complaints of right loin pain and hematuria. The patient's medical history was notable for hypertension, which had been managed with lisinopril 10 mg once daily for the past eight years. Prior to this medication, her blood pressure had been controlled through dietary and lifestyle modifications, including salt restriction and exercise. There was no significant surgical history. The patient was G1P1, with one living offspring. She is a homemaker and does not smoke or consume alcohol. Her father had hypertension, but otherwise, her family history was unremarkable.

On admission, her vitals were within normal range, including a blood pressure of 135/90 mmHg, a pulse rate of 88 beats per minute, and a temperature of 36.7°C. Abdominal examination showed only right flank tenderness without a palpable mass. Otherwise, the physical examination was unremarkable, and there were no abnormalities found during the neurological examination.

**Fig. 1:**
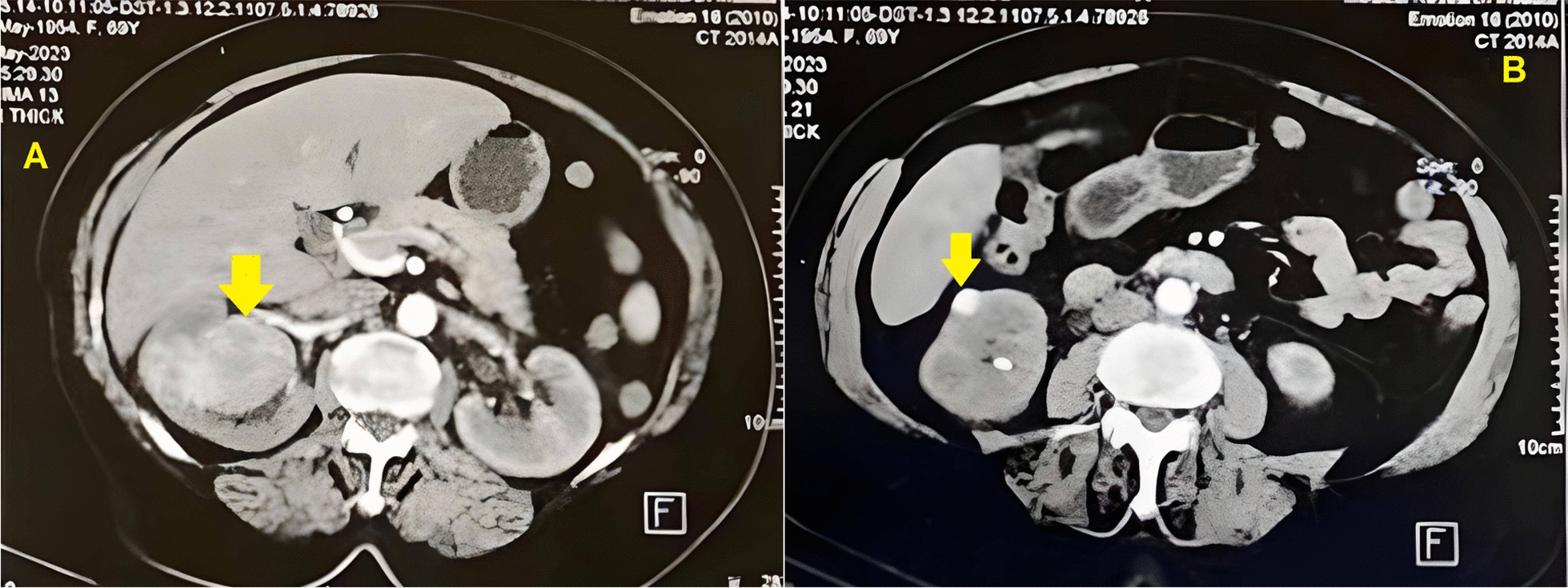
Multi-slice computed tomography displaying two right renal masses. **A** The first mass was an upper polar mainly anterior, totally endophytic, enhancing soft tissue mass lesion measuring 6.5 cm and invading the middle calyx. **B** The second smaller mass was a lower polar exophytic lesion measuring 1.3 cm

The lab results showed anemia, indicated by a hemoglobin level of 9.5 g/dL, and a normal serum creatinine level of 0.7 mg/dL [6, 7]. Liver function tests were within normal ranges, and urinalysis showed trace protein and numerous red blood cells without casts. Multi-slice computed tomography (MSCT) revealed two right renal masses. The first one was an upper polar mainly anterior, totally endophytic, enhancing soft tissue mass lesion measuring 6.5 cm and invading the middle calyx with a RENAL score [8] of 11A (Fig. 1A). The other smaller mass was a lower polar exophytic lesion measuring 1.3cm (Fig. 1B). An apparent left paraaortic lymph node measuring 10 × 8.3 mm was detected. Multiple calyceal filling defects at the delayed phase suggesting blood clots were also reported. Metastatic workup was free.

**Fig. 2:**
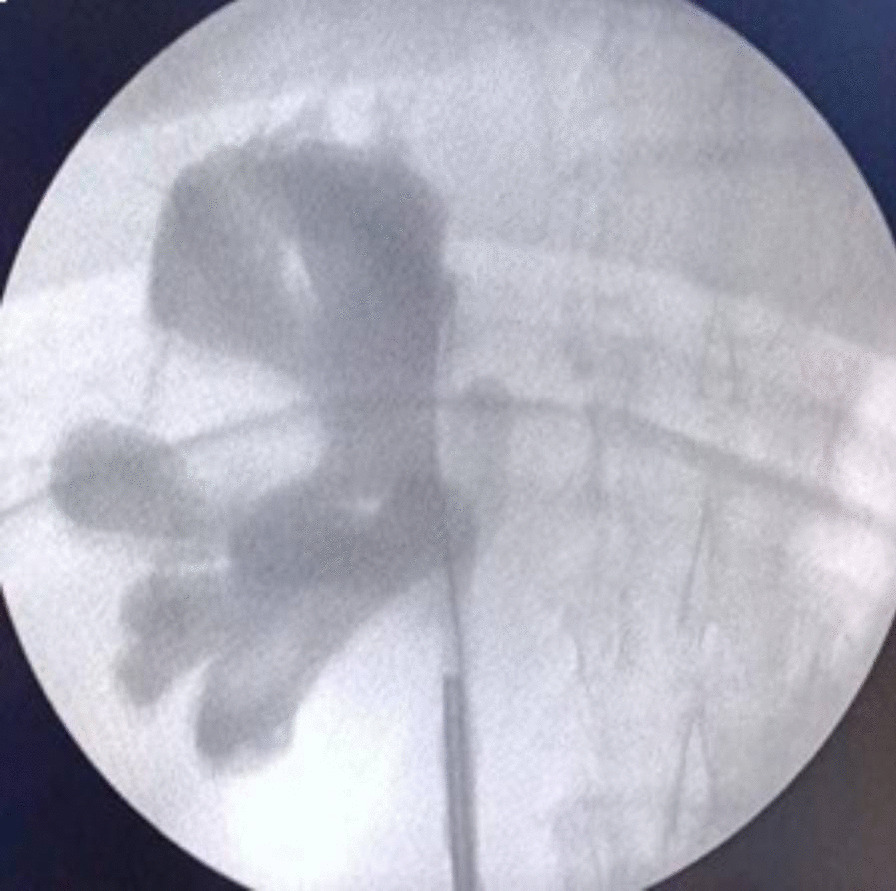
Intraoperative right retrograde contrast study showing no filling defect of the pelvicalyceal system. This right retrograde contrast study was performed since the patient originally presented with hematuria. After no pelvicalyceal filling defect was detected, the surgical team proceeded to the right laparoscopic radical nephrectomy

Considering the history of hematuria, diagnostic cystoscopy, and right retrograde contrast study were done intraoperatively to exclude upper tract urothelial tumor (Fig. 2), the latter showing no filling defect of the pelvicalyceal system. The patient then underwent laparoscopic right radical nephrectomy with a presumptive clinical diagnosis of multicentric neoplastic renal process, mostly RCC.

**Fig. 3:**
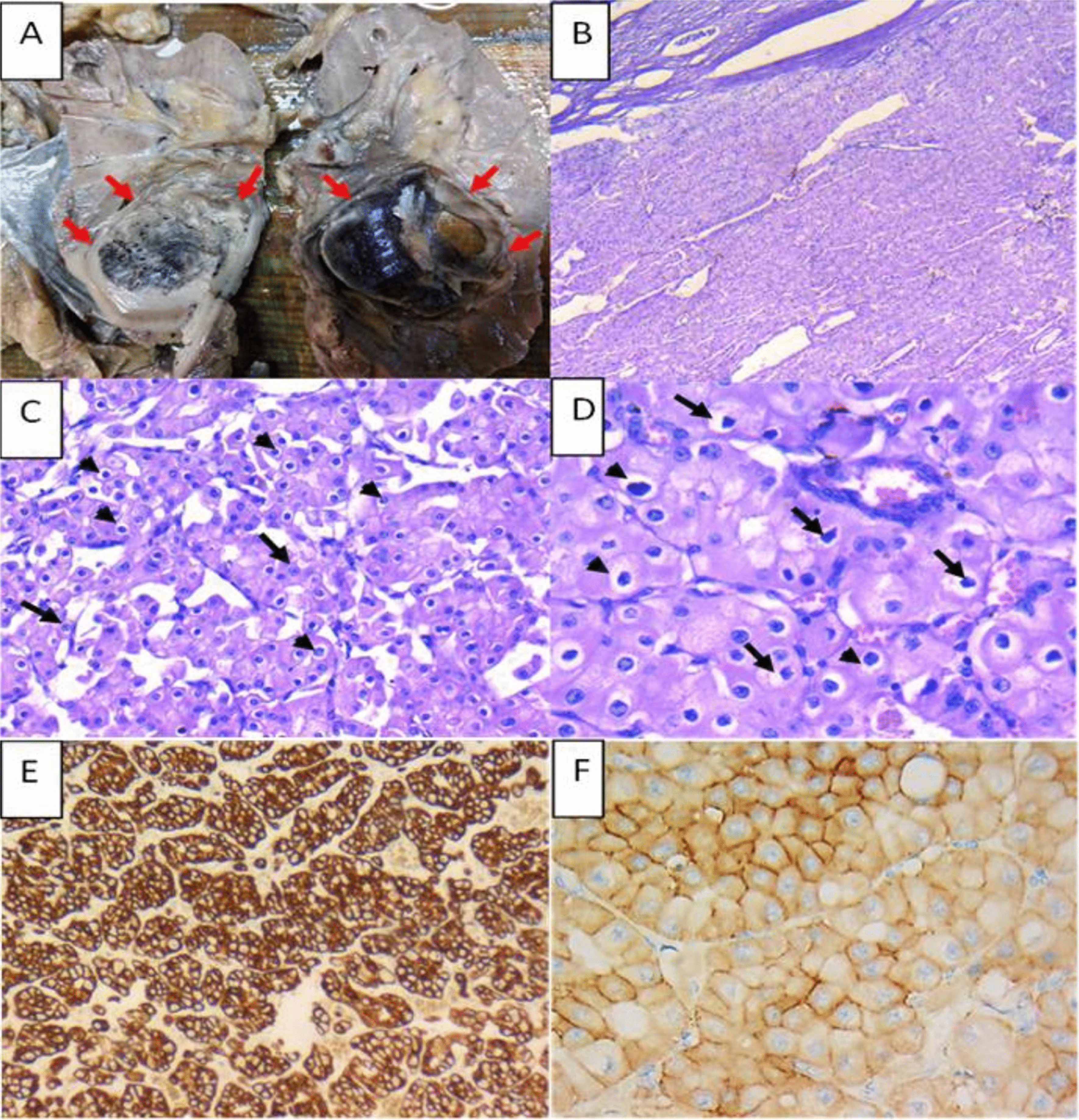
Pathological gross and microscopic examination of the larger, upper polar right renal mass (chromophobe renal cell carcinoma). **A** Macroscopic examination revealed a well-circumscribed non-encapsulated mass having a hemorrhagic brown cut section extending from the upper pole to the midzone of the kidney. **B** Low power examination revealed a well-circumscribed neoplastic growth formed of sheets of oncocytic cells with focal tubular pattern (H & E: × 40). **C** The tumor cells have well-defined cell borders and eosinophilic cytoplasm. Their nuclei are rounded to oval with occasional prominent nucleoli (arrows), perinuclear halos are also seen (arrowheads) (H &E, × 200). **D** The neoplastic cells are arranged in trabeculae; they have abundant eosinophilic cytoplasm and round to oval nuclei with occasional raisinoid nuclei (arrows) and perinuclear halos (arrowheads). (H &E, × 400). **E** The tumor cells are strongly positive for CK-7 (CK-7, × 100), and **F** The tumor cells are positive for CD117 (CD117, × 200)

Serial sectioning of the specimen grossly revealed two distinct well-circumscribed masses. The larger mass extended from the upper pole to the midzone of the kidney and measured 4.6 × 4 × 4 cm. Its cut surface was solid hemorrhagic brown with few yellowish areas. The tumor did not invade the renal sinus, renal vein, pelvicalyceal system, or perinephric fat grossly (Fig. 3A). Microscopic examination of the larger mass revealed a well-circumscribed nonencapsulated neoplastic growth formed of sheets and trabeculae of polygonal cells having abundant eosinophilic cytoplasm and well-defined cell outlines with plant-like cell borders. The cells had round to oval nuclei, showing mild to moderate pleomorphism with occasional prominent nucleoli. Irregular raisinoid nuclei with irregular nuclear membranes and occasional perinuclear halos were also noted (Fig. 3B–D). No necrosis, sarcomatoid, or rhabdoid features were detected. The tumor was morphologically diagnosed as ChRCC, confirmed by immunohistochemical positivity for CK-7 and CD117 (Fig. 3E and F).

**Fig. 4:**
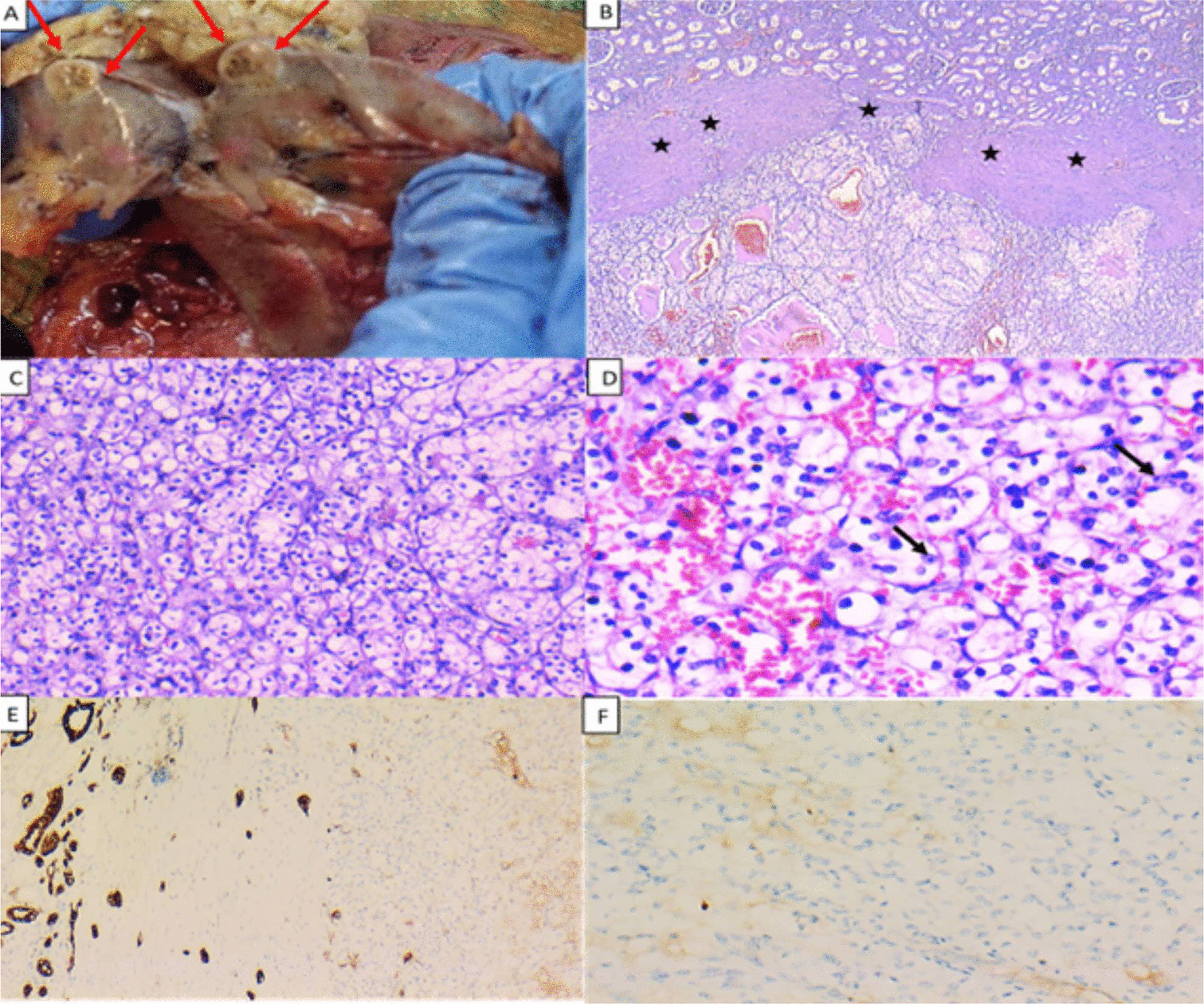
Pathological gross and microscopic examination of the smaller, lower polar right renal mass (clear cell renal cell carcinoma). **A** Macroscopic examination revealed a partially exophytic mass at the lower pole of the kidney (arrows). The mass is light brown and shows minute cystic spaces. **B** The tumor is formed of clear cells arranged as nests and tubules (lower part of the image) separated from the non-neoplastic renal parenchyma (upper part of the image) by a fibrous pseudo-capsule (asterisks) (H &E: × 40). **C** Nests of tumor cells separated by thin stroma (H &E: × 200). **D** The neoplastic cells have clear cytoplasm, and mildly pleomorphic nuclei, with occasional conspicuous nucleoli (nuclear grade 2) (arrows). Note the evident intervening richly vascular septae between tumor cells (H & E, × 400). **E** The tumor cells are negative for CK-7 (Right) with positive internal control detected in the normal renal tubules (left) (CK-7, × 100), and the tumor cells are negative for CD117 (CD117, × 200) (**F**)

The second smaller mass was located at the lower pole of the kidney, it measured 1.3 × 1 × 0.8 cm and was partially exophytic. Its cut section was light brown with minute cystic spaces. Again, no invasion of the renal sinus, renal vein, pelvicalyceal system, or perinephric fat was detected grossly (Fig. 4A). Histopathological examination revealed a different histologic subtype of renal cell carcinoma forming a well-circumscribed neoplastic growth and having a fibrous pseudo-capsule. It was formed of clear cells arranged in solid sheets as well as nests / alveolar pattern separated by delicate vascular spaces. Focally, a tubular pattern was also seen. The cells had clear cytoplasm and mildly pleomorphic nuclei with conspicuous nucleoli at high power examination. No necrosis, rhabdoid, or sarcomatoid features were noted (Fig. 4B-D). This tumor was morphologically diagnosed as ccRCC, with a nuclear grade of 2 (according to the WHO/ISUP grading system) and was confirmed by negative immunohistochemical staining of the neoplastic cells for both CK-7 and CD117 (Fig. 4E and F). Both tumors did not invade the renal sinus, renal vein, renal pelvis, or perinephric fat microscopically. One unit of packed red blood cells was administered intraoperatively.

After surgery, the patient received 500 mL of isotonic saline every 12 h for one day and intravenous ceftriaxone 1 gm every 12 h for five days, along with paracetamol as needed for pain management. Her postoperative recovery was favorable, with a repeat hemoglobin level of 10.4 g/dL and serum creatinine level of 0.8 mg/dL. No specific adjuvant therapy was prescribed, and she was discharged one week later with prescriptions for levofloxacin 750 mg once daily for two weeks and ibuprofen tablets for pain relief as needed.

She was scheduled for 3 monthly follow-up visits. During the 3-month and 6-month follow-up appointments, laboratory and imaging tests, including complete blood count, renal function tests, and computed tomographic (CT) studies of the chest, abdomen, and pelvis were all unremarkable.

## Discussion

The coexistence of two histologically distinct renal cell carcinomas (RCC) within the same kidney is an exceedingly rare phenomenon. Most documented instances of synchronously coexisting neoplasms with RCC in the literature have involved benign neoplasms [1–5]. However, our case presents a unique occurrence involving a combination of chromophobe RCC (ChRCC) and clear cell RCC (ccRCC) simultaneously occurring in the same kidney. To the best of our knowledge, this is the ninth reported case of such a combination in the literature. Furthermore, unlike many cases reported in the literature where intraoperative detection was the case, the multifocal RCC in our patient was successfully identified through preoperative imaging.

RCC constitutes 85% of all renal neoplasms. It arises from the renal tubular epithelium and is frequently asymptomatic upon diagnosis [5, 9]. RCCs are categorized into clear-cell, papillary, chromophobe, collecting duct, and unclassified carcinomas [10]. Clear cell (ccRCC) is the most common subtype, accounting for 75% of RCC cases, followed by papillary (pRCC) at 10% and chromophobe (ChRCC) at 5% [2, 11]. Accurately distinguishing between these RCC subtypes is vital and essential to guarantee the most appropriate treatment plan and prognosis for the patient [5].

The coexistence of RCC in the same kidney with other neoplasms, whether benign or malignant, is rare, and the presence of different RCC histologic subtypes together is even more exceptionally uncommon [1–5]. The most frequently reported combinations include RCC with oncocytoma, angiomyolipoma (rarely without tuberous sclerosis), or even an adrenal adenoma [12]. The synchronous presence of multiple dissimilar histologic subtypes of RCC is even rarer and can present as two spatially separate, histologically distinct RCCs or a single discrete tumor composed of two types of RCC tumor components.

Many theories were proposed to explain the phenomenon of multiple yet different RCCs coexisting: the theory of cancer stem cells (CSCs) provides a potential explanation for the emergence of two or three dissimilar renal tumors [2, 4, 9]. Other hypotheses are the evolution of one subtype into another [2], as oncocytomas transforming into pRCC [13]**,** or the infiltration of an aggressive tumor into another tumor, as collecting duct carcinoma (CDC) infiltrating pRCC [4].

We constituted a thorough literature review of reports of cases with synchronous dissimilar RCC histologic subtypes in Table 1. We believe that our case adds to the literature on the topic. Our patient is considered the 9th case presenting with the combination of ccRCC and ChRCC in the literature. We also discussed the authors’ approach to preoperative detection and differentiation, selection of operative technique, and postoperative follow-up problems in terms of difficulties in estimating prognosis and unclear follow-up outcomes in the literature.

**Table 1:** Previous studies reporting cases of synchronous yet dissimilar RCCs

Author	Number of patients, n	Histology combinations, n^d^	Presenting symptom, n^d^	Procedure done	Side	Site^e^	Other renal tumors
Kuroda *et al*. [21]^a^	1	ccRCC; ccRCC; cc-pRCC	N/A	Radical nephrectomy	Left	Upper; lower; middle	
Kang *et al*. [5]	1	ChRCC; ccRCC	Left flank pain; huge palpable mass	Radical nephrectomy	Left	Upper; middle	AMLs
Tyritzis *et al*. [2]	1	ChRCC; pRCC	Painless microscopic hematuria	Radical nephrectomy	Right	Upper; lower	
Roehl *et al*. [9]^b^	1	ChRCC; pRCC	N/A	Radical nephrectomy	Left	Upper	
Delto *et al*. [1]^a^	1	ChRCC; ccRCC; pRCC	vague abdominal pain	Partial nephrectomy	Right	Interpolar; upper; lower	
Na *et al*. [10]	1	pRCC; ccRCC		Radical nephrectomy			
Harlow *et al*. [3]^c^	15	ccRCC; ChRCC, 3 pRCC; cc-pRCC, 2 ccRCC; cc-pRCC, 2 ccRCC; pRCC, 2 pRCC; acquired cystic disease RCC, 2 pRCC; unclassified RCC, 1 ccRCC; pRCC, 3		Partial or radical nephrectomy			
Capaccio *et al*. [16]	5	ccRCC; pRCC, 3 ccRCC; ChRCC, 1 pRCC; ChRCC, 1	Macrohematuria, 2 Lumbar pain, 1 Asymptomatic, 2				
Lee *et al*. [22]	1	ccRCC; ChRCC				Midportion; lower	
Jun *et al*. [23]	1	ChRCC; ccRCC		Radical nephrectomy	Left	Midportion; lower	AML
Arik *et al*. [4]^b^	1	pRCC; CDC	Flank pain; hematuria	Radical nephrectomy	Right	Lower	
Renshaw *et al*. [19]	2	ChRCC; pRCC, 2				N/A	
Auget *et al*. [24]	1	ccRCC; CDC			Right	Upper; lower	
Daniel *et al*. [25]	1	pRCC; CDC			Right	Upper	
Gong *et al*. [26]^b^	1	ChRCC; CDC			Left	Lower	
Lindgren *et al*. [27]^b^	1	ChRCC; CDC			Right	Lower	
Cho *et al*. [28]^b^	1	ccRCC (unclassified); CDC			Left		
Matei *et al. *[29]	1	pRCC; CDC			Left	Upper; medulla	
Kawano *et al*. [30]^b^	1	ChRCC; CDC			Left	Middle; lower	
Tsai *et al*. [31]	1	ccRCC; CDC					
Quiroga-Garza *et al*. [32]	1	ccRCC; tubulocystic			Right	Upper; mid-lateral	
Esposito *et al*. [33]	5						

*ccRCC* clear cell renal cell carcinoma, *cc-pRCC* clear cell papillary renal cell carcinoma, *ChRCC*  chromophobe renal cell carcinoma, *N/A*  not available, *AML*  angiomyolipoma, *CDC*  collecting duct carcinoma, *RMICT*  renomedullary interstitial cell tumor

^a^Cases with 3 synchronous RCCs; ^b^cases with 1 tumor displaying multiple different histologic morphologies of RCC; ^c^3 out of 15 patients had a single tumor that showed multiple different histologic RCC subtypes; ^d^Histologic combinations and presenting symptoms in number of patients, n, are stated if the study has more than one patient; ^e^Site of each tumor is mentioned respectively to the order in which histology is stated

Regarding differentiation using histological features under light microscopy, ccRCC is characterized by a solid to alveolar growth pattern with abundant vascular networks and occasional tubule formation, with cytoplasm of tumor cells varying from clear to eosinophilic granular. These findings were in accordance with those found in our ccRCC. ChRCC is identified by polygonal cells forming sheets and trabeculae with eosinophilic granular cytoplasm, with occasional irregular raisinoid nuclei, irregular nuclear membranes, and perinuclear halos, again consistent with our findings in the ChRCC. Furthermore, electron microscopy revealed the presence of numerous characteristic small cytoplasmic vesicles in the chromophobe areas [9].

In our case, the larger tumor was ChRCC and showed positive immunohistochemical staining for CK7 and CD117, while the smaller tumor was ccRCC and displayed negative immunohistochemical staining for both CK-7 and CD117. These results are in accordance with the reported immunohistochemical profile of both tumors in literature [14, 15].

Preoperative knowledge of the coexistence of multiple RCC subtypes in the same kidney is important not only for planning the surgical approach, especially if nephron-sparing surgery is opted for, but also for postoperative determination of prognosis, since not all RCC subtypes are of similar aggressive tendencies, and applicability of possible neoadjuvant regimens.

Imaging, thus, is a cornerstone tool in our armamentarium to determine this coexistence. In a report by Capaccio *et al*., ultrasound (US) was able to recognize the presence of multiple renal tumors in 1 of 5 cases versus CT in 4 of 5 cases [16]; the lower number of cases in which multiplicity of renal lesions is detected by the US may be explained by the lower sensitivity of US in detecting small renal nodules than that of CT [17]**.** Although CT has proven useful in detecting multifocal lesions, its ability to do so is limited. As a result, it is still relatively common to discover unsuspected multifocal disease during surgery. Capaccio *et al*. also concluded that different enhancement patterns on CT may enable the preoperative detection of tumors with different histology within the same kidney [16]; however, distinguishing different renal tumors on CT presents challenges, as the image is influenced not only by the main histologic type but also by factors such as the degree of differentiation, size, and presence of necrosis. Our MSCT successfully identified a multifocal neoplastic renal process, revealing two lesions: a 6.5 cm predominantly anterior endophytic upper polar lesion and a 1.3 cm exophytic lower polar lesion.

Treatment strategies for renal tumors vary based on the patient's overall condition, as well as tumor characteristics, including size, position, relationship, number, and aggressiveness [16]. In terms of aggressiveness, clear-cell renal cancers have the highest malignant potential and a 5-year survival rate of 50–60%, while papillary and chromophobe renal cell carcinomas are associated with lower metastatic potential and an overall 5-year survival of 80–90% [18, 19].

While most literature favors radical nephrectomy for treating synchronous dissimilar coexisting RCCs, recent studies have shown that patients with sporadic single or multiple ipsilateral renal tumors may opt for nephron-sparing surgery, which yields comparable oncological results with low morbidity and recurrence rates [20]. Our patient underwent radical nephrectomy, with negative margins for both tumors and no invasion of the renal pedicle or Gerota's fascia.

Due to the relatively short follow-up periods (typically 3–6 months) in case reports of patients with multifocal synchronous RCC found in the literature, estimating prognosis can be challenging, with insufficient information about long-term outcomes [5]. The presence of multifocal pathology may increase the risk of recurrence for the patient [1]. Additionally, the various subtypes of renal tumors can influence disease-free survival after radical nephrectomy, making it difficult to assign patients to specific adjuvant therapy protocols when two separate and distinct carcinomas are present in a nephrectomy specimen [5]. Our patient showed no involvement of the renal arterial and venous pedicle, suprarenal glands, Gerota's fascia, or distant organs on preoperative imaging. In addition, her follow-up plan, consisting of a CT chest, abdomen, and pelvis and laboratory estimation of hemoglobin and serum creatinine levels every three months, was unremarkable. However, estimating the prognosis is still challenging due to the limited information on follow-up in the literature. In addition, our case was not assigned adjuvant chemotherapy or radiotherapy, reflecting the difficulty in selecting specific adjuvant regimens when multiple dissimilar RCCs coexist also reported in the literature [5].

## Conclusion

Given the scarcity of reports, challenges in preoperative detection and differentiation, the possible intraoperative discovery of multifocality, and the dilemma in selecting appropriate adjuvant regimens for cases with multiple dissimilar RCCs, further research on this topic is urgently needed. This research should focus on enhancing preoperative detection and differentiation of multiple renal lesions, planning optimal operative techniques (radical or partial nephrectomy), selecting suitable adjuvant regimens, and reporting long-term follow-up outcomes. Since the management, prognosis, and long-term survival of different histological subtypes of RCC may be different, accurate recognition of the possible coexistence of these tumors is extremely critical and mandates multidisciplinary involvement, including meticulous preoperative imaging by radiologists and proper, thorough grossing of nephrectomy specimens by pathologists.

## Data Availability

Data included in the manuscript.

## Abbreviations

RCC: Renal cell carcinoma
ccRCC: Clear cell renal cell carcinoma
ChRCC: Chromophobe renal cell carcinoma
MSCT: Multi-slice computed tomography
pRCC: Papillary renal cell carcinoma
CSCs: Cancer stem cells
CDC: Collocting duct carcinoma
US: Ultrasound

## References

[1] Delto J, Hussein SE, Fukuma B, Abello A, Alexis J, Mastrogiovanni L (2018). Multifocal synchronous chromophobe, papillary, and clear cell renal cell carcinoma in a single kidney. J Urol Surg..

[2] Tyritzis SI, Alexandrou PT, Migdalis V, Koritsiadis G, Anastasiou I (2009). Synchronous chromophobe and papillary renal cell carcinoma. First report and review of the pathogenesis theories. Pathol Int..

[3] Harlow BL, Klaassen Z, Holzman SA, Reinstatler L, Franken AA, Kavuri S (2016). Multiple discordant histology after nephrectomy: descriptive analysis and outcomes. Clin Genitourin Cancer..

[4] Arık D, Açıkalın M, Can C (2015). Papillary renal cell carcinoma and collecting duct carcinoma combination. A case report and review of synchronous renal cell carcinoma subtypes in the same kidney. Arch Med Sci AMS..

[5] Kang S, Ko Y, Kang S, Kim J, Kim C, Park H (2010). Two different renal cell carcinomas and multiple angiomyolipomas in a patient with tuberous sclerosis. Korean J Urol.

[6] Ceriotti F, Boyd JC, Klein G, Henny J, Queralto J, Kairisto V (2008). Reference intervals for serum creatinine concentrations: assessment of available data for global application. Clin Chem.

[7] Turner J, Parsi M, Badireddy M. Anemia. StatPearls. Treasure Island (FL) ineligible companies. StatPearls Publishing. Copyright © 2023, StatPearls Publishing LLC.; 2023.

[8] Parsons RB, Canter D, Kutikov A, Uzzo RG (2012). RENAL nephrometry scoring system: the radiologist’s perspective. Am J Roentgenol.

[9] Roehrl MHA, Selig MK, Nielsen GP, Dal Cin P, Oliva E (2007). A renal cell carcinoma with components of both chromophobe and papillary carcinoma. Virchows Arch.

[10] Na K, Kim HS, Park YK, Chang SG, Kim YW (2012). Multifocal renal cell carcinoma of different histological subtypes in autosomal dominant polycystic kidney disease. Korean J Pathol..

[11] Li G, Cottier M, Sabido O, Gentil-Perret A, Lambert C, Genin C (2005). Different DNA Loidy patterns for the differentiation of common subtypes of renal tumors. Cell Oncol.

[12] Yin G, Zheng S, Zhou C, Li Y (2023). Concurrent angiomyolipoma and chromophobe renal cell carcinoma in the same kidney: a case report. Asian J Surg.

[13] Al-Saleem T, Balsara B, Liu Z, Feder M, Testa J, Wu H (2005). Renal oncocytoma with loss of chromosomes Y and 1 evolving to papillary carcinoma in connection with gain of chromosome 7. Coincidence or progression?. Cancer Genet Cytogenet..

[14] Zhao W, Tian B, Wu C, Peng Y, Wang H, Gu W-L (2015). DOG1, cyclin D1, CK7, CD117 and vimentin are useful immunohistochemical markers in distinguishing chromophobe renal cell carcinoma from clear cell renal cell carcinoma and renal oncocytoma. Pathol Res Pract.

[15] Kim M, Joo JW, Lee SJ, Cho YA, Park CK, Cho NH (2020). Comprehensive immunoprofiles of renal cell carcinoma subtypes. Cancers.

[16] Capaccio E, Varca V, Simonato A, Toncini C, Carmignani G, Derchi LE (2009). Synchronous parenchymal renal tumors of different histology in the same kidney. Acta Radiol.

[17] Jamis-Dow C, Choyke P, Jennings SB, Linehan W, Thakore KN, Walther M (1996). Small (< or = 3-cm) renal masses: detection with CT versus US and pathologic correlation. Radiology..

[18] Cheville J, Lohse C, Zincke H, Weaver A, Blute M (2003). Comparisons of outcome and prognostic features among histologic subtypes of renal cell carcinoma. Am J Surg Pathol..

[19] Renshaw A, Henske E, Loughlin K, Shapiro C, Weinberg D (1996). Aggressive variants of chromophobe renal cell carcinoma. Cancer.

[20] Krambeck AE, Iwaszko MR, Leibovich BC, Cheville JC, Frank I, Blute ML (2008). Long-term outcome of multiple ipsilateral renal tumours found at the time of planned nephron-sparing surgery. BJUI..

[21] Kuroda N, Shiotsu T, Kawada C, Shuin T, Hes O, Michal M (2011). Clear cell papillary renal cell carcinoma and clear cell renal cell carcinoma arising in acquired cystic disease of the kidney: an immunohistochemical and genetic study. Ann Diagn Pathol..

[22] Lee T, Kim W, Lee S, Kang K, Jeong Y, Kang M (2010). Double synchronous primary renal cell carcinoma with different histotypes. Clin Nephrol.

[23] Jun S-Y, Cho K-J, Kim C-S, Ayala AG, Ro JY (2003). Triple synchronous neoplasms in one kidney: report of a case and review of the literature. Ann Diagn Pathol..

[24] Auguet T, Molina JC, Lorenzo A, Vila J, Sirvent JJ, Richart C (2000). Synchronus renal cell carcinoma and Bellini duct carcinoma: a case report on a rare coincidence. World J Urol.

[25] Daniel L, Zattara-Cannoni H, Lechevallier E, Pellissier J (2001). Association of a renal papillary carcinoma with a low grade tumour of the collecting ducts. J Clin Pathol.

[26] Gong Y, Sun X, Haines GK, Pins MR (2003). Renal cell carcinoma, chromophobe type, with collecting duct carcinoma and sarcomatoid components. Arch Pathol Lab Med.

[27] Lindgren V, Paner GP, Flanigan RC, Clark JI, Campbell SC, Picken MM (2004). Renal tumor with overlapping distal nephron morphology and karyotype. Arch Pathol Lab Med.

[28] Cho N, Kim S, Ha M, Kim HJ (2004). Simultaneous heterogenotypic renal cell carcinoma: immunohistochemical and karyoptic analysis by comparative genomic hybridization. Urol Int..

[29] Matei D-V, Rocco B, Varela R, Verweij F, Scardino E, Renne G (2005). Synchronous collecting duct carcinoma and papillary renal cell carcinoma: a case report and review of the literature. Anticancer Res.

[30] Kawano N, Inayama Y, Nakaigawa N, Yao M, Ogawa T, Aoki I (2005). Composite distal nephron-derived renal cell carcinoma with chromophobe and collecting duct carcinomatous elements. Pathol Int..

[31] Tsai T-H, Tang S-H, Chuang F-P, Wu S-T, Sun G-H, Yu D-S (2009). Ipsilateral synchronous neoplasms of kidney presenting as acute pyelonephritis and bladder metastasis. Urology.

[32] Quiroga-Garza G, Piña-Oviedo S, Cuevas-Ocampo K, Goldfarb R, Schwartz MR, Ayala AG (2012). Synchronous clear cell renal cell carcinoma and tubulocystic carcinoma: genetic evidence of independent ontogenesis and implications of chromosomal imbalances in tumor progression. Diagn Pathol.

[33] Esposito M, Varca V, Simonato A, Toncini C, Carmignani G, Derchi L (2009). Coexistence of different histotypes of renal carcinoma: our experience and literature review. Rivista Urol..

